# Seroprevalence Estimates of Q Fever and the Predictors for the Infection in Cattle, Sheep, and Goats in Nandi County, Kenya

**DOI:** 10.1155/2022/3741285

**Published:** 2022-11-16

**Authors:** J. Kiptanui, P. B. Gathura, P. M. Kitala, B. Bett

**Affiliations:** ^1^Department of Public Health, Pharmacology and Toxicology, University of Nairobi, Faculty of Veterinary Medicine, P.O. Box 29053-00625, Nairobi, Kenya; ^2^International Livestock Research Institute, P.O. Box 30709-00100, Nairobi, Kenya

## Abstract

Q fever is an important worldwide zoonotic disease that affects almost all domestic animals, wildlife, and humans. The infection has both socio-economic and public health significance. A cross-sectional study was carried out to investigate the estimates of seroprevalence of Q fever and to determine the predictors of the infection in cattle, sheep, and goats in six wards of Nandi County. A total of 1,140 blood samples were collected from 366 households. Samples were drawn from 725 cattle (64%), 283 sheep (25%), and 132 goats (11%). Multistage sampling method was adopted. Serum samples were analyzed for antibodies to *Coxiella burnetii* using the indirect ELISA test. Results showed an overall animal seroprevalence of 5.614% (64/1140) for Q fever. In cattle, the seroprevalence was 8.138% (59/725) with CI 95% (2.8–18.23), 1.413% (4/283) for sheep CI 95% (1.0–7.78), and 0.758% (1/132) goats CI 95% (0.14–7.27). From the findings, Q fever was more prevalent in cattle (OR 7.26) than in sheep and goats. Animal species (*p* value 0.015, CI 95% OR 7.26) was the only potential predictors in the three considered species for the presence of *Coxiella burnetii* antibodies. Sex, age, breed, and production system had no statistical significant association for Q fever infection since *p* value was >0.005. In conclusion, the results demonstrated that cattle, sheep, and goats are widely exposed to Q fever organisms, and hence, it is an important zoonosis in Nandi County. Therefore, to address this “silent” disease, there is an urgent call for both veterinarians and medical personnel to jointly address prevention and control strategy through enhanced surveillance, public sensitization, and awareness creation under the one health concept. There is also a need for enhanced capacity for the diagnosis of Q fever in both animals and humans in Nandi County.

## 1. Introduction

Q fever is a zoonotic disease caused by the obligate intracellular bacterium *Coxiella burnetii.* It is an important human and veterinary public health problem worldwide [1]. The organism infects mammals, birds, reptiles, and arthropods. It causes a mild disease in ruminants with the common symptoms being abortions, stillbirths, weak off springs, metritis, and infertility. Coxiellosis is regarded as endemic worldwide except in New Zealand [2]. In humans, Q fever is mostly asymptomatic. The acute form is mainly limited to flu-like illness, pneumonia, or hepatitis whereas the chronic form manifests with chronic fatigue or endocarditis. In pregnant women, *C. burnetii* infections are known to cause abortions, stillbirth, and premature deliveries [3]. The greatest risk of transmission of *C. burnetii* organisms occurs at parturition by inhalation, ingestion, or direct contact with birth fluids or placenta. The infected animal shed the bacterium through the placenta and birth fluids which may contaminate the environment leading to airborne dissemination and infection of persons in close contact with livestock [4].

In many developed countries, Q fever is considered a reportable disease, but in most African countries, the infection is not part of the priority list of diseases under surveillance by the concerned authorities (VSD [5]). The main reason is the scanty data on the epidemiology of Q fever. This, therefore, implied that Q fever could be missed out in differential diagnosis to be considered in abortion and infertility cases in livestock and in flu-like and febrile conditions in men. Although C. burnetii infection has been detected in humans and in a wide range of animal species across the continent, seroprevalence varies widely by species and location. Studies on animal seroprevalence in most African countries revealed infection by C. burnetii among cattle was ≤13% except for studies in Western and Middle Africa which reported 18–55%. Small ruminant seroprevalence ranged from 11 to 33%. Human seroprevalence was ≤8% with the exception of studies among children in Egypt (10–32%) [6].

In Kenya, Q fever is regarded as an old and neglected zoonotic disease in a wide range of animal and human populations. Despite the fact that the disease is widely distributed, it is underdiagnosed and underreported because of its diverse symptoms, self-limiting course, and lack of diagnostic tools [7]. The factors influencing pathogen transmission, persistence, and spread are poorly understood. According to [8], Q fever infection is believed to contribute to undiagnosed reproductive and febrile diseases in livestock and the farming communities. The epidemiology of Q fever in Kenya is poorly understood due the apparent neglect of the disease by both medical and veterinary personnel [9]. Strategies such as integrated disease surveillance and prevention/control programs are urgently required. A number of studies done earlier appeared to have been mainly in arid and semiarid lands, only inhabited by pastoralists [10]. The information on the same in high-potential areas was scarce or completely lacking in some regions. In Nandi County, there was no available data on Q fever infection in ruminants, despite being home to over 309,038 exotic dairy breeds and their crosses, 121,461 sheep, and 46,669 goats [11]. The current study is the first to be undertaken in the County.

The prevalence rate from surveyed areas in Kenya ranged from 7.4 to 51.1% in cattle, 6.7–20.0% in sheep, 20.0–46.0% in goats, and 20.0–46.0% in camels [12]. According to Koka et al. [13], the prevalence of Q fever was reported at 12.1% in both livestock and human population in five of the seven former provinces of Kenya surveyed, and the risk was reportedly higher in grazed animals. In Baringo County, the seroprevalence of Q fever was 26.0% and 12.2% in goats and sheep, respectively [14]. Despite these findings, the information on Q fever and its risk factors was lacking in Nandi County and most high-potential regions of central and south Rift valley where dairy is the main source of livelihood. This study was, therefore, carried out to establish the actual estimates of seroprevalence and associated risk factors of Q fever in cattle, sheep, and goats in Nandi County, Kenya.

## 2. Materials and Methods

### 2.1. Study Area

The study was conducted in Nandi County, which is located in the North Rift of Kenya, occupying an area of 2,884.4 square kilometres. According to the Kenya national bureau of statistics (KNBS) [11] national census, the County had a human population of 885,711 made up of a number of Kenyan communities, the majority of whom belong to the native Nandi community. The County borders Kakamega County to the west and south-west, Uasin Gishu County to the north and north-east, Kericho County to the south-east, Kisumu County to the south, and Vihiga County to the south and south-west. Geographically, the jug-shaped structure of Nandi County is bound by the equator to the south and extends northwards. It lies within latitudes 0° and 0° 34″ North and longitudes 34° 45″ and 35° 25″ East. The county lies at an altitude ranging between 1300 and 2500 metres above sea level. It has a cool and moderately wet climate and receives an average rainfall of between 1200 mm and 2000 mm per annum. Most parts of the county experience mean temperatures of between 18°C and 22°C.

**Figure 1:**
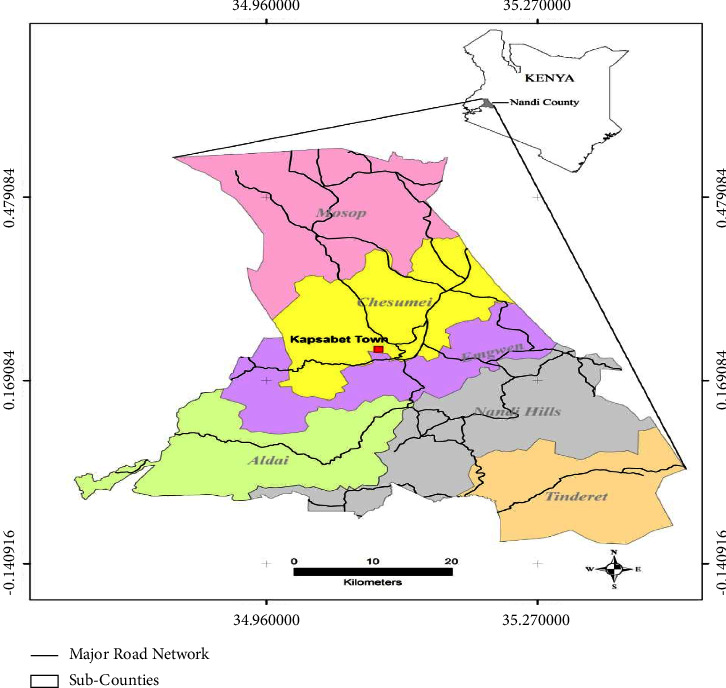
Map of Kenya showing the location of Nandi county and the six subcounties (2019).

Administratively, the county comprises of 6 subcounties, namely, Mosop, Chesumei, Emgwen, Aldai, Nandi Hills, and Tindiret with a total of 30 wards. Agriculturally, it is a high-potential area with dairy and crop farming as the main activity. In terms of Q fever, the county was perceived as a high-risk area as a result of the large livestock population and the socio-cultural practices of living in close proximity with the livestock [15]. Below is the map of Kenya showing the location of Nandi County (Figure 1).

### 2.2. Study Design

A cross-sectional survey was undertaken between June 2019 and September 2019 to collect blood samples for the estimation of seroprevalence of Q fever and the questionnaire to determine the predictors of bacterium infection in cattle, sheep, and goats.

### 2.3. Sample Size

Sample size determination was done according to the formula in Dohoo et al. 2010.

(1) $$\begin{array}{c} n=\frac{Z_{a^{2}}pq}{L^{2}}\text{,} \end{array}$$

where *Z* is the value of the normal deviate that produces 95% confidence intervals, *p* is a priori estimate of the bacterium from other studies [16] *p*=6.8% for cattle, 6.6% for goats and 4.9% for sheep, and *L* is the standard error of the estimate. Thus, a total of 725 cattle above 12 months of age with no history of previous vaccination against Q fever were sampled for this study including 283 sheep and 132 goats.

### 2.4. Sampling

A multistage sampling was used to select the study animals. In the first stage, the six subcounties were selected namely Chesumei, Mosop, Emgwen, Nandi Hills, Aldai, and Tindiret. In the secondary stage, six wards were selected from the six subcounties by simple random sampling, and these included Lelmokwo/Ngechek ward in Chesumei, Kabisaga ward in Mosop, Kilibwoni ward in Emgwen, Lessos ward in Nandi Hills, Kaboi/Kaptumo ward in Aldai, and Tindiret ward in Tindiret. In the third stage, households were selected randomly, and a total of 366 households were included in the study. Herds within households were systematically selected until the required number of sample size was achieved.

### 2.5. Data Collection

Data were collected by administration of semistructured and pretested questionnaires via personal interviews with the head of the household or a representative. Data collected included household demographics and potential predictors were animal species, sex, age, breed, and production systems.

### 2.6. Blood Collection

Approximately 10 ml of blood sample was collected aseptically from the jugular vein of each animal with the disposable needle into a vacutainer tube. The tubes were labeled, placed in a rack, and put in a cool box with enough ice. They were transported to the Regional Veterinary Investigation laboratory (RVIL) Eldoret within 12 hours after collection for sera processing.

### 2.7. Laboratory Analysis

Blood samples were kept in the refrigerator (2–8°C) overnight, and serum was separated by centrifuging. The sera samples were put in cryovials, labeled, and preserved at −20°C at the facility after which they were transported to International Livestock Research Institute (ILRI), Nairobi for analysis using indirect enzyme-linked immunoabsorbent assay (iELISA).

The ELISA kit for Q fever was sourced from IDEXX Laboratories, Inc, USA. The Q fever antibodies test used was an enzyme immunoassay for the detection of antibodies against *Coxiella burnetii* in serum, plasma, and milk samples of ruminants. The testing protocol for iELISA for *C burnetii* was done according to the manufacturer's instructions. Briefly, antibodies to C. burnetii were detected by the commercial indirect ELISA test using a 96-well microtiter plates precoated with the C. burnetii phase I and II strains. Positive and negative control sera were included in each plate. Serially diluted sera in phosphate-buffered saline containing 0.1% Tween 20 was added and then incubated. After incubation, the plates were washed to remove any unbound material. Antibodies are detected with alkaline phosphatase-conjugated rabbit anti-human IgG, IGM, and IgA at optimal dilution. Both antiphase I and II antibodies are detected. Positive and negative control sera were included in each plate. Color developed in the presence of bound enzyme and the optical density was read with an ELISA plate reader. As recommended by the manufacturer, an animal was considered to be ELISA-strong positive if the optical density (OD) was over 80%. An OD between 50% and 80% was considered positive. A doubtful ELISA result was noted if the OD was between 40% and 50%, while an OD ≤ 40% was considered a negative animal. The sensitivity and specificity of the ELISA test kit as provided by the manufacturer were 99% and 98%, respectively.

### 2.8. Data Handling and Analysis

Data collected were entered into the Microsoft Excel spreadsheet programme (Microsoft Corp) for editing and cleaning. The data were then imported to SPSS statistical package version 20 (SPSS Inc., Chicago, IL, USA, 2002). Tables of descriptive statistics were generated including means, modes, and proportions. Tests of association were done at two levels. In the univariate logistic regression models, the independent variables and the dependent variables were analyzed for the association. The significance for the univariate analysis was set at *p* ≤ 0.05. Those variables that were significant in the univariate analysis were used to develop multivariate logistic models using a backward elimination procedure. Results were displayed in tables and graphs.

### 2.9. Ethical Considerations

Ethical approval was sought before the research commenced from the University of Nairobi, Faculty of Veterinary Medicine Biosafety, Animal Care, and Use Committee. Permission was requested and granted by the County government of Nandi to undertake the research. Consent was sought from participants, and identities remained confidential.

## 3. Results

**Figure 2:**
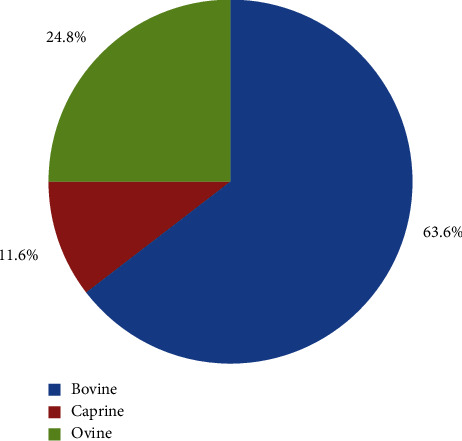
Distribution of blood samples collected per species in Nandi county, Kenya (2019).

**Figure 3:**
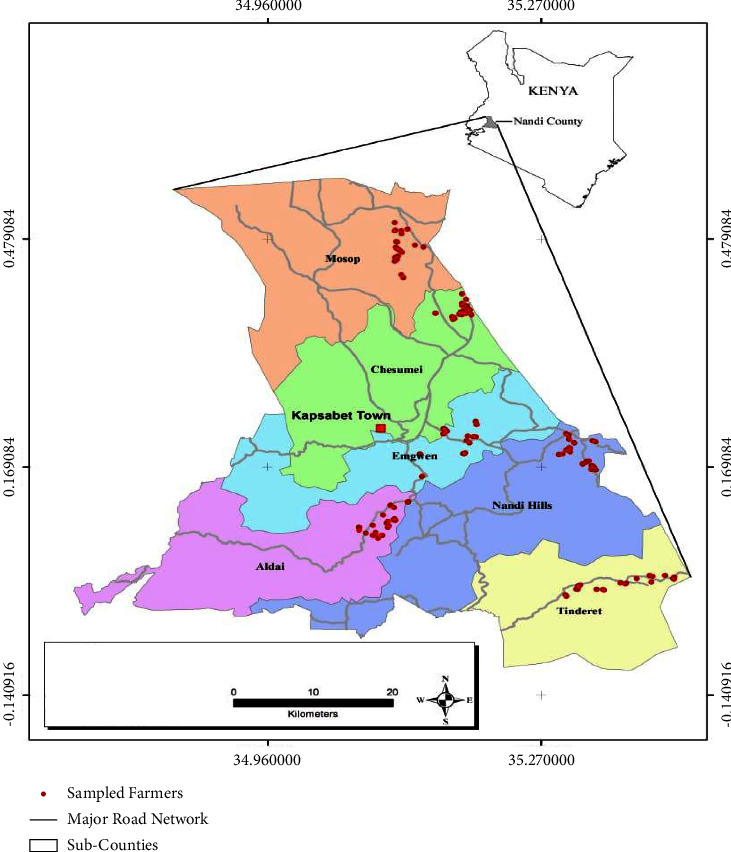
Spatial distribution of blood samples collected in Nandi county, Kenya (2019).

A total of 1,140 blood samples were collected from cattle 63.6% (725/1140), sheep 24.82% (283/1140), and goats 11.58% (132/1140). These data were collected from 366 households in six wards, one from each of the six subcounties in Nandi County. The distribution of the collected blood samples per species and the sampling sites is shown in Figures 2 and 3, respectively.

### 3.1. Seroprevalence of Q Fever in Cattle, Sheep, and Goats in Nandi County

From the total blood samples (1,140) collected, sixty-four (64) serum samples tested positive for *C. burnetii* antibodies. These findings showed that overall animal seropositivity in the County was 5.614% (64/1140). Moreover, seropositivity in cattle was 8.138% (59/725) OR 7.26 and CI 95% (2.8–18.23), sheep 1.413% (4/283) OR 0.028 at CI 95% (1.0–7.7), and goats 0.758% (1/132) OR 0.11 and CI 95% (0.14–7.27). Results confirmed that Q fever was present in ruminants in the County. From this observation, Q fever seropositivity was more in cattle than sheep and goats (Table 1). These findings demonstrated that the likelihood of cattle getting infected with Q fever was seven times (OR 7.26) more than sheep and goats.

### 3.2. Predictors of Q Fever Seropositivity in Cattle, Sheep, and Goats in Nandi County

The risk factors for Q fever in animals considered were animal species, sex, breed, age, and production systems. Below is the distribution of animal species testing positive for Q fever as per the six study sites. Emgwen Sub-County had the highest seroprevalence rate (11.54%) in cattle, followed by Tindiret (10.87%), and the lowest was Nandi hills Sub-Counties (4.62%). The above is illustrated in Table 2.

### 3.3. Seroprevalence of Q Fever Based on Sex, Age, and Breed Categories

Q fever prevalence was highest in male caprine at 9.09% (1/11), followed by female bovines at 8.48% (57/672) and male bovines at 3.85% (2/52). The prevalence for female sheep was 1.70% (4/236), but 0% for both male sheep and female goats. In terms of breed, Arshyire and Jersey recorded the highest figures of 15.23% (37/243) and 8.70% (2/23), respectively. According to the age category, adult bovines' recorded higher seropositivity of 8.33% (49/588) compared to heifers 6.67% (9/135) (Table 3).

### 3.4. Seroprevalence of Q Fever Based on Production System Category

In Nandi County, cattle, sheep, and goats were raised under four production systems, namely extensive, semiintensive, zero grazing, and tethering. Results pointed out that the prevalence of Q fever was higher in zero grazing units 12.12% (4/33) compared to semiintensive production system 8.24% (48/582), extensive production system 2.8% (10/357), and tethering 2/154 (1.29%).

### 3.5. Association of Q Fever Seroprevalence with Species, Sex, Age, Breeds, and Production System

A multivariate logistic regression was adopted to measure the effects of the independent variables (species, sex, breed, and production system) on the dependent variable (Q fever). Following the analysis, the only variable that was statistically associated with the seropositivity of Q fever was animal species. From the result, cattle were more prone to Q fever infection 8.138% (59/725) compared to sheep 1.413% (4/283) and goats 0.758% (1/132) (OR 7.26, *p* value 0.015 CI 95%) (Table 4).

The other variable analyzed was sex, where results demonstrated Q fever was more common in female than male animals. From the observation, 61 out of 64 positive cases were females, and 3 were male animals. Indeed in cattle, females recorded a higher figure 57/672 (8.482%) and were two times (OR 2.32) more likely to be positive than sheep and goats. These findings however had no statistical significance to show a positive association between Q fever and sex variables (*p* value 0.065). Also, the test of association amongst the breeds and age categories of the species under investigation did not yield any statistical significance with Q fever infection (*p* value >0.05), likewise to the production system (*p* value >0.05).

## 4. Discussion

According to the Nandi County integrated development plan [17], the livestock subsector is regarded as the key driver for socio-economic activity and supports the livelihood of many rural poor households. Dairy cattle, sheep, and goats are the main livestock species kept in Nandi County. Dairy farming is the heart of the community's livelihood since apart from getting food and income; they have a special sentimental attachment to their animals. The majority of the farmers practiced mixed farming with maize, tea, and sugarcane as the major commercial crops. According to Muturi et al. [18], zoonotic diseases are a public health priority with Rift valley, Brucellosis, and Q fever as the three top priority diseases in the country.

This study was the first in the County to investigate the status of Q fever disease in cattle, sheep, and goats and its associated risk factors. According to Njeru et al. [12], Q fever infections impart socio-economic burden due to production and reproductive losses such as abortions, stillbirths, and infertility. This research was, therefore, undertaken to establish the true status of Q fever infections in Nandi given that the County is endowered with over 300,000 cattle of which the majority are exotic and their crosses (Friesian, Ayrshire, Jersey, Guernsey), 120,000 sheep (Dorper and Merino), and 45,000 goats (Galla goats and Toggenburg). The findings showed that the seropositivity in cattle was 8.138% (59/725), 1.413% (4/283) sheep, and 0.758% (1/132) goats. Despite the status of the disease being unknown, these results were an eye-opener because they proved the wide spread of Q fever infection in ruminants, hence a significant threat to human health especially amongst people living in close proximity to the animals. The results further showed the disease was more prevalent in cattle followed by sheep and goats, respectively. The findings were in agreement with the previous study where seroprevalence of Q fever in cattle was estimated at 7.4–51.1% and 8.77% in Kenya [12] and Ethiopia [19], respectively. However, in small ruminants, these results differed where the prevalence was lower than the reported figure of 20–46% in goats and 6.7–20% in sheep. Furthermore, other surveys done in the neighboring countries reported higher *C. burnetii* seropositivity than the current study in domestic animals. For example, in Tanzania, it reported 13.3% in cattle, 13.6% in goats, and 17.1% in sheep [20]. Sudan reported seropositivity of 24% in goats, 40.4% in cattle, 53% in goats, and 62.5% in sheep [21]. In another region of Ethiopia, it was 31.6% in cattle, 54.2% in goats, and 90% in camel [22].

In Kenya, the seroprevalence of Q fever was higher (28.2–57.1%) in livestock from pastoralist communities [10, 23] than in highlands. Though the precise reason for this variation is not clear, agroecological zones, animal production systems, livestock, and human density patterns appear to play a key role. In pastoral communities, practices such as the mixing of large numbers of animals, movement of livestock in search of pasture, sharing of grazing areas with wildlife, and concentration of animals around water points are linked to the higher transmission of Q fever among cattle, goats, and sheep. Seroprevalence in highlands tends to be lower because they are well managed in confined individual farms with the majority of farmers using artificial insemination or own bull/ram/buck for breeding purposes. Other reasons included minimal movements, limited shared resources, and animals rarely come in contact with wildlife. This could be true in the current study because Nandi County is generally wet and cold and farming systems are sedentary with minimum movement of livestock and shared resources. Also, animals are kept in enclosed individual farms with rare wildlife interaction.

In this study, the following risk factors in animals were investigated: species, sex, age, breed, and production systems. However, animal species was the only identified risk factor associated with Q fever infection. It showed *C. burnetii* seroprevalence was higher in cattle (8.138%) than in sheep 1.413% and goats (0.758%). It further demonstrated that cattle were 7 times more prone to Q fever compared to the other species (OR 7.26). Data on the epidemiology of Q fever were scarce, particularly in terms of the source of infection. But according to Ioannou, [24]; he documented that ticks are linked to the spread of Q fever where they acted as a reservoir of the pathogen. This could be true in Nandi County where ticks may be playing a key role in Q fever transmission because 80% of diseases reported yearly are tick-borne diseases mainly East coast fever, anaplasmosis, and babesiosis among others [25]. Therefore, further studies on the role of ticks in the transmission of coxiellosis in Nandi County may be required to ascertain this assumption.

In terms of sex, the overall seropositivity of Q fever was 5.94% (61/1027) in females and 0.29% (3/1027) in males. Male goats were leading with 9.091% (1/11), followed by female cows at 8.482% (57/672) and ewes at 3.482% (4/236). There were no positives in does and rams. These results differed from a previous study done in Ghana where both sexes were infected in equal measure irrespective of the number sampled [26]. Despite these findings, the current study, however, confirmed sex was not a risk factor for the Q fever infection.

The results further illustrated that adult cows had a higher seropositivity rate (4.737%) compared to heifers (0.877%). These findings were in agreement with a study done by Mwololo [23] in Bura, Tana River County, where he reported low seropositivity in young animals. A possible explanation could be due to more exposure to *C. burnetii* antibodies. On breed characteristics, there was no significant difference in the positivity of Q fever in the County. In this study, Jersey breed had a slight high prevalence of 8.696% as compared to Ayrshire and Friesian which recorded a prevalence of 8.230% and 8.259% respectively. Toggenburg breed in goats had a percentage prevalence of 3.846%, and the Dorper breed in sheep was 1.379%. This finding may be associated with genetics and exposure to the causative agent. In terms of the production system, the seroprevalence of Q fever showed that zero grazing and semi-intensive production systems had a higher prevalence rate of 8.889% and 8.233%, respectively, compared to 2.801% and 1.299% for the extensive and tethering. The findings corresponded to a previous study done by Ibrahim et al. [27] which reported extensive production systems had lower Q fever seropositivity as compared to semi-intensive and zero grazing. The possible explanation could be associated with contamination related to hygiene challenges in intensive production system particularly the management of animal wastes. Despite these results, multivariate analysis found no significant statistical association between Q fever and these independent variables since *p* value was >0.05 (sex, age, breed, and farming production systems).

Lastly, the gap observed in this study which needs to be addressed was that Q fever was not among the priority list of diseases under surveillance by the County veterinary authority, coupled with a lack of diagnostic facility in the region that has the capacity to test for Q fever. This, therefore, implied that Q fever could be missed out in differential diagnosis to be considered in abortions, stillbirths, and infertility cases in livestock.

## 5. Conclusion

The present study provided valuable data on the seroprevalence of Q fever in cattle, sheep, and goats in Nandi County and its association with different risk factors. Detection of Q fever presence demonstrated that cattle, sheep, and goats are exposed to *C. burnetii* antibodies in Nandi County. The results further showed a high seroprevalence of Q fever in cattle as compared to sheep and goats which confirmed that the infection is an important zoonosis in the County with significant public health and socio-economic importance. Despite the disease and its status being unknown in the County, the results were an eye opener that revealed Coxiellosis was widely spread and could be one of the reasons for missed diagnosis of abortions and infertility in animals. We recommended farmers' and stakeholders' sensitization and awareness to enhance participation in disease surveillance and control program for better control and prevention of Q fever in Nandi County. Also, there was a need for enhanced capacity for the diagnosis of Q fever in both animals and humans.

## Figures and Tables

**Table 1:** Summary of seroprevalence of Q fever in cattle, sheep, and goats in Nandi county.

Animal	Total sample	iELISA positive	% Seropositivity
Cattle	725	59	8.138
Sheep	283	4	1.413
Goats	132	1	0.758
Total	1,140	64	5.614

**Table 2:** Distribution of animal species testing positive for *C. burnetii* antibodies on iELISA as per sub-county in Nandi County.

Sub-County	Livestock species								
	Cattle			Sheep			Goats		
	No. Tested	No. Positive	Prevalence (%)	No. Tested	No. Positive	Prevalence (%)	No. Tested	No. Positive	Prevalence (%)
Aldai	128	12	9.38	61	2	3.28	0	0	0
Chesumei	143	12	8.39	44	1	2.27	6	0	0
Emgwen	104	12	11.54	66	0	0	2	0	0
Mosop	128	7	5.47	44	0	0	0	0	0
Nandi hills	130	6	4.62	64	1	1.56	3	0	0
Tindiret	92	10	10.87	4	0	0	121	1	0.83
Total	725	59	8.138	283	4	1.413	132	1	0.758

**Table 3:** Distribution of Q fever seropositivity according to species, sex, breed, and age.

Univariate logistic regression model													
Species	Sex			Breed					Age				Seropositive animals
Bovine	Female	Male	N/S	Ayrshire	Friesian	Guernsey	Jersey	Zebu	Adults	Heifers	Yearlings	N/S	
	57/672 (8.48%)	2/52 (3.85%)	1	37/243 (15.23%)	20/448 (4.46%)	0/4 (0%)	2/23 (8.7%)	0/6 (0%)	49/588 (8.33%)	9/135 (6.67%)		2	59/725 (8.14%)

Caprine				Galla	Saanen	Toggenburg							
	0/119 (0%)	1/11 (9.09%)	2	0/94 (0%)	0/3 (0%)	1/26 (3.85%)			1/123 (0.81%)		0/9 (0%)		1/132 (0.76%)

Ovine				Doper	Merino								
	4/236 (1.7%)	0/47 (0%)	0	4/290 (1.38%)	0/2 (0%)				4/246 (1.63%)		0/37 (0%)		4/283 (1.41%)

Total samples	1140			1140					1140				64/1140 (5.61%)

**Table 4:** Multivariate logistic regression model on Q fever and animal species.

Dependent variable	Independent variable	*N*	Proportion positive (%)	Odd ratios (OR)	Seropositivity (%)	CI 95%	*p* values
Q fever	Bovines	725	59 (8.138)	7.26	8.14%	2.8–18.23%	0.015
	Caprine	132	1 (0.758)	0.11	0.76%	0.14–7.27%	0.317
	Ovine	283	4 (1.413)	0.028	1.41%	1.0–7.78%	0.076

## Data Availability

The data that support the findings from this study were part of the research done for the attainment of a degree in Doctor of Philosophy of Veterinary Public Health and will be available at the University of Nairobi repository. The author further confirms that the data from this study will also be available in Veterinary Medicine International Journal as a research article.
